# The complete mitochondrial genome of *Artemia persimilis* Piccinelli and Prosdocimi, 1968 (Crustacea: Anostraca)

**DOI:** 10.1080/23802359.2022.2036258

**Published:** 2022-03-09

**Authors:** Xuekai Han, Lahm Tashi, Liying Sui, Guishuang Wang, Gusang Deji, Chi Zhang

**Affiliations:** a Asian Regional Artemia Reference Center, Tianjin University of Science and Technology, Tianjin, China; b Institute of Fisheries Science, Tibet Academy of Agricultural and Animal Husbandry Sciences, Lhasa, P. R. China; c Tibet Academy of Agricultural and Animal Husbandry Sciences, Lhasa, P. R. China

**Keywords:** *Artemia persimilis*, mitochondrial genome, phylogenetic relationship

## Abstract

In the study, we report the complete mitochondrial genome of *Artemia persimilis* Piccinelli and Prosdocimi, 1968 for the first time. The mitochondrial genome of *A. persimilis* is 15,436 bp in length, with the typical structure of 13 protein-coding genes (PCGs), 22 transfer RNA genes (tRNAs) and 2 ribosomal RNA genes, and a non-coding control region (CR). Phylogenetic analysis showed that *A. persimilis* was at the basal position among the bisexual *Artemia* species, which revealed that *A. persimilis* is likely to be an ancestral clade. The present study could provide effective resources for population genetics study, as well as germplasm conservation in *Artemia*.

## Introduction

*Artemia* is not only one of the most important live food items used in larval aquaculture, but also an ideal laboratory model organism for scientific research. As a small crustacean, *Artemia* have a widely distribution all over the world, mainly in hypersaline environments, and play an important biological regulatory role in salt field ecosystem. The genus *Artemia* is generally considered to contain seven bisexual species as well as some parthenogenetic *Artemia* populations with different polyploidy types (Asem et al. 2010). However, their generic taxonomy is not universally accepted, especially in relation to *Artemia tibetiana* and *Artemia urmiana*. Mitochondrial DNA are widely used to study the molecular ecology of animals because it is convenient and economical, comparison of the mitochondrial genomes will permit examination of evolution and relationships between species. At present the complete mitochondrial genome of four bisexual species (*Artemia franciscana*, *A. urmiana*, *A. tibetiana* and *Artemia sinica*) is already known (Valverde et al. 1994; Zhang et al. 2013; Asem et al. 2019). But for another bisexual species *Artemia persimilis* Piccinelli and Prosdocimi, 1968, a species endemic to the South American and geographically restricted to Argentina and Chile (Sainz Escudero et al. 2021), the complete mitochondrial genome has not been reported and characterized. Herein, we reported the complete mitochondrial genome sequence of *A. persimilis*, and we performed a phylogenetic analysis to study the evolutionary relationships of *A. persimilis* with other *Artemia* species. We expect that the present result will facilitate the further investigations of phylogenetic relationship, taxonomic resolution and phylogeography of the *Artemia* species.

## Materials and methods

The cysts of *A. persimilis* Piccinelli and Prosdocimi, 1968 was collected from Bahia Blanca, Argentina (**latitude** −62.1338 and **longitude** −38.8385). The specimen was deposited at the Asian Regional Artemia Reference Center (Tianjin University of Science and Technology, China) (Liying Sui, suily@tust.edu.cn) under the voucher number 1807. The genomic DNA was extracted form cysts according to the instructions of TIANGEN®TIANamp Genomic DNA Kit (Tianjin, China). Then the high quality gDNA was sequenced using Illumina Novaseq6000 platform with 350 bp insert size. The complete mitochondrial genome was assembled using SPAdes v.3.5.0 (http://cab.spbu.ru/software/spades/) with *A. franciscana* (GenBank accession number: X69067) as reference. The reference mitochondrial map and BLAST (https://blast.ncbi.nlm.nih.gov/Blast.cgi) were used for gene annotation. The tRNA genes were predicted using the ARWEN (http://mbio-serv2.mbioekol.lu.se/ARWEN/) and tRNAscan-SE 2.0 (http://lowelab.ucsc.edu/tRNAscan-SE/) online software.

## Results and discussion

The complete mitochondrial genome of *A. persimilis* Piccinelli and Prosdocimi, 1968 (Genbank assession number: MZ199176) is 15,436 bp in length, with the typical structure of 13 protein-coding genes (PCGs), 22 transfer RNA genes (tRNAs) and 2 ribosomal RNA genes, and a non-coding control region (CR). The base composition is 25.70% A, 24.00% C, 20.07% G, and 30.23% T, with an A + T content of 55.93%. Just six PCGs (*cox1*, *atp6*, *cox3*, *cytb*, *nd1* and *nd2*) began with the common ATG start codon. Stop codons included TAA (*cox3*, *nd2*, *atp8*, *atp6*, *nd3* and *cytb*) and TAG (*nd1*, *nd4l* and *nd6*). There are 4 genes ended with the incomplete stop codon T (*cox1*, *cox2*, *nd4* and *nd5*). The 12S rRNA and 16S rRNA were separated by the *trnV*. The length of *rrnL* and *rrnS* is 1148 bp and 711 bp, respectively. The 22 tRNA genes size varies from 61 to 67 bp, respectively.

**Figure 1.**
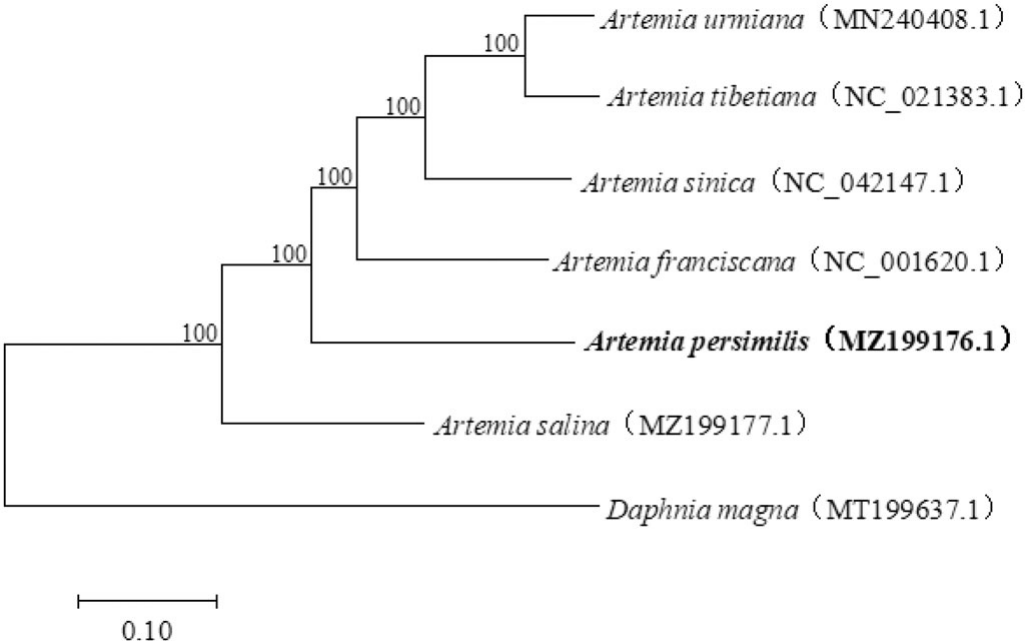
Phylogenetic tree showing the relationship among *A. persimilis* and other species from the *Artemia*. The numbers on each node are the bootstrap support values. *Daphnia magna* was selected as an outgroup.

Combined with the complete mitochondrial genome sequences of four bisexual *Artemia* species as well as diecious *Artemia salina* from GenBank, a phylogenetic tree was constructed by Maximum-likelihood (ML) method with the Kimura 2-parameter model using the software MEGA 7.0 (Kumar et al. 2016), so as to infer the phylogenetic relationships among *Artemia* species. The phylogenetic tree showed that *A. persimilis* was at the basal position among the bisexual *Artemia* species, which revealed that *A. persimilis* is likely to be an ancestral clade (Figure 1). The mitochondrial genome sequence of *A. persimilis* reported here provide a useful genetic resource for population genetics and evolutionary studies in *Artemia*.

## Data Availability

The data that support the findings of this study are openly available in GenBank of NCBI at https://www.ncbi.nlm.nih.gov, reference number MZ199176. The associated BioProject, SRA, and BioSample numbers are PRJNA749897, SRR15292985, and SAMN20424866, respectively.
